# Navigating the Therapeutic Landscape of Multiple Myeloma: Immunotherapy, Microenvironment, and Resistance

**DOI:** 10.3390/biomedicines14071556

**Published:** 2026-07-11

**Authors:** Sreejeta Mondal, Nathan Becker, Yang Huo, Pengyue Zhang, Travis S. Johnson, Carl Ola Landgren, David G. Coffey, Brian A. Walker, Enze Liu

**Affiliations:** 1 Myeloma Institute, Sylvester Comprehensive Cancer Center, Miller School of Medicine, University of Miami, Miami, FL 33136, USA; sxm3595@miami.edu (S.M.); njbecker@miami.edu (N.B.); col15@miami.edu (C.O.L.); davidcoffey@miami.edu (D.G.C.); brianwalker@miami.edu (B.A.W.); 2 Department of Biomedical Informatics, College of Medicine, Ohio State University, Columbus, OH 43210, USA; huo.116@osu.edu; 3 Department of Biostatistics & Health Data Sciences, School of Medicine, Indiana University, Indianapolis, IN 46202, USA; zhangpe@iu.edu (P.Z.); johnstrs@iu.edu (T.S.J.)

**Keywords:** multiple myeloma, tumor microenvironment, bispecific antibodies (BiTEs), chimeric antigen receptor T-cell (CAR-T) therapy, antibody–drug conjugates (ADCs), toxicity, resistance

## Abstract

Immunotherapies, including chimeric antigen receptor (CAR) T cells, bispecific T-cell engagers (BiTEs), and antibody–drug conjugates (ADCs), have revolutionized the treatment landscape for multiple myeloma (MM). Despite robust initial response rates, achieving durable remissions remains challenging due to frequent relapses driven by complex therapeutic resistance mechanisms. In this review, we comprehensively examine intrinsic tumor resistance, such as innate and acquired antigen escape mediated by genomic alterations, structural variations, and epigenetic silencing. Furthermore, we highlight the critical role of the highly permissive bone marrow microenvironment in blunting the efficacy of modern therapies. Cellular compartments, including mesenchymal stromal cells, osteoclasts, and expanded immunosuppressive immune populations, actively foster tumor survival, promote metabolic competition, and T-cell exhaustion. We also review the unique clinical toxicities associated with T-cell-redirecting modalities, including cytokine release syndrome (CRS) and immune effector cell-associated neurotoxicity syndrome (ICANS). Ultimately, deciphering the complex interplay between malignant plasma cells and their surrounding microenvironment is essential for optimizing treatment sequencing, preventing effector cell exhaustion, and designing next-generation therapeutic strategies to secure long-term, durable responses for patients.

## 1. Introduction

Multiple myeloma is a complex hematological malignancy characterized by the proliferation of malignant plasma cells within the bone marrow [1,2,3]. The clinical evolution of multiple myeloma (MM) typically advances from a precursor state, known as monoclonal gammopathy of undetermined significance (MGUS), through smoldering myeloma and eventually into active myeloma [4]. This progression is marked by a profound remodeling of the bone marrow microenvironment [1]. Malignant plasma cells interact with surrounding cellular compartments, including mesenchymal stromal cells, osteoclasts, and various immune cells, to create a highly permissive niche [3,5]. This dynamic crosstalk not only fuels tumor growth and bone lesions but also drives the expansion of immunosuppressive cell populations, such as myeloid-derived suppressor cells (MDSCs) and regulatory T cells (Tregs), resulting in a loss of effective immune surveillance [5,6,7].

Historically, multiple myeloma treatment relied on foundational agents such as alkylating agents and proteasome inhibitors. Immunomodulatory drugs (IMiDs) enhance T-cell and natural killer (NK)-cell activity while inhibiting tumor angiogenesis [7,8]. Monoclonal antibodies targeting SLAMF7 and CD38 then emerged as highly effective options that significantly improved response rates [9,10,11,12]. Recently, the therapeutic landscape has undergone a paradigm shift driven by the identification of cell surface proteins that are highly expressed on malignant plasma cells, such as B-cell maturation antigen (BCMA), GPRC5D and FcRH5 [12,13,14,15,16,17,18,19]. The development of targeted immunotherapies directed at these antigens—including chimeric antigen receptor (CAR) T cells, bispecific T-cell engagers (BiTEs), and antibody–drug conjugates (ADCs)—has significantly improved clinical outcomes and achieved robust initial response rates, even in heavily pretreated populations.

Despite these advances, multiple myeloma remains an incurable disease characterized by frequent relapses, presenting a critical unmet need [20,21]. Treatment failure in a substantial proportion of patients arises due to either intrinsic tumor resistance, such as innate or acquired antigen escape, or extrinsic factors within the bone marrow microenvironment such as severe metabolic competition and the induction of T-cell and NK-cell exhaustion [22,23]. Furthermore, toxicities and systemic infections from emerging T-cell-redirecting therapies require rigorous clinical management [24,25]. Consequently, there is an urgent need to delineate these resistance mechanisms and microenvironmental interactions to optimize treatment sequencing and secure long-term, durable responses in patients.

In this review, we provide a comprehensive overview of the evolving immunotherapeutic modalities in multiple myeloma and how they are influenced by microenvironmental factors to drive immune evasion, therapeutic resistance, and treatment-associated toxicities, while highlighting emerging strategies to overcome these challenges.

## 2. The Malignant Bone Marrow Microenvironment: A Permissive Niche

**Figure 1:**
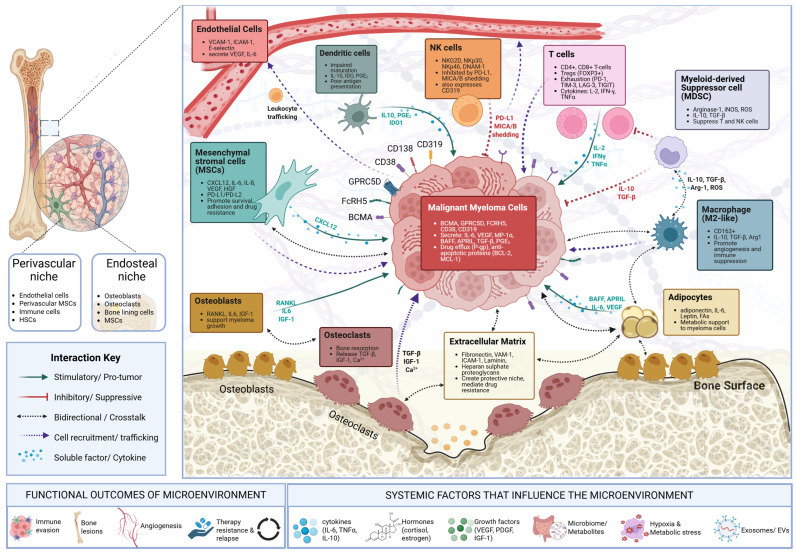
Summary of surface proteins, signaling molecules and receptors on various cells in the bone marrow microenvironment.

The bone marrow microenvironment represents a highly specialized and dynamic niche that supports hematopoiesis and immune homeostasis. It can be broadly divided into two anatomical niches—the perivascular niche and the endosteal niche—which together maintain normal immune and blood cell production along with healthy bone structure [1]. The perivascular niche sits along the marrow blood vessels, especially sinusoidal vessels and is composed primarily of endothelial cells and perivascular stromal cells that support hematopoietic stem and progenitor cells through local signaling interactions. The endosteal niche, on the other hand, is the bone-surface compartment lined by osteoblasts and other osteo-lineage cells along the endosteum. This niche primarily harbors quiescent long-term repopulating hematopoietic stem cells (HSCs). Collectively, these niches form a dynamic bone marrow microenvironment composed of diverse cellular compartments—including mesenchymal stromal cells (MSCs), osteoblasts, osteoclasts, endothelial cells, immune, non-immune, and hematopoietic cells as well as acellular components comprising the extracellular matrix and a ‘liquid milieu’ rich in cytokines and growth factors that reciprocally respond to diverse signaling cues [2,3]. In MM, this tightly regulated environment is co-opted by tumor cells to promote tumor growth, immune evasion, and therapeutic resistance (Figure 1).

### 2.1. Mesenchymal Stromal Cells (MSCs)

Mesenchymal stromal cells (MSCs) are a major non-hematopoietic component of the bone marrow stroma and regulate immune cell trafficking and bone remodeling. They also support hematopoietic stem cell self-renewal and differentiation. Under normal physiological conditions, MSCs maintain immune homeostasis by modulating anti-inflammatory and immunosuppressive responses through secretion of cytokines that polarize macrophages toward anti-inflammatory M2 phenotypes, suppress T-cell proliferation, and promote regulatory T cells [5,26]. In the myeloma bone marrow microenvironment, MSCs acquire enhanced immunosuppressive properties and secrete soluble factors like PD-1 ligands (PD-L1 and PD-L2), which suppress T-cell activation and induce immune tolerance. Expression of these ligands is further induced by inflammatory cytokines like IFN-γ and TNF-α [27]. In myeloma, crosstalk between MSCs and myeloma cells plays a critical role in tumor proliferation, survival, migration, and therapeutic resistance. MSCs derived from myeloma patients are genetically, transcriptionally, and functionally distinct from normal donor-derived MSCs, exhibiting reduced osteogenic differentiation, impaired hematopoietic niche support, and altered immunosuppressive properties that allow their persistence even in the absence of tumor cells [28,29,30,31].

### 2.2. Immunosuppressive Cells

Immune cells derived from hematopoietic stem and progenitor cells (HSPCs) largely mature within the bone marrow, except for T cells. Following immune activation in peripheral tissues, a subset of these cells returns to and persists in the bone marrow, highlighting its dual role as both a primary hematopoietic organ and a secondary lymphoid site [32]. The bone marrow niche regulates HSC maintenance and differentiation through specialized cellular and molecular interactions that coordinate immune cell development and sustain hematopoietic homeostasis [33].

The progression from MGUS to MM is marked by remodeling of the bone marrow microenvironment, characterized by loss of effective immune surveillance and expansion of immunosuppressive populations such as MDSCs, Tregs, regulatory B cells (Bregs), and tumor-associated macrophages (TAMs) [34]. These cells secrete inhibitory cytokines including IL-10 and TGF-β, as well as mediators such as nitric oxide and reactive oxygen species, while STAT3 activation further promotes IL-6 and VEGF production and supports Treg development through FOXP3 signaling [35]. MDSCs are significantly increased in both the peripheral blood and bone marrow of patients with active MM compared with healthy donors. Their expansion is associated with disease progression and poor clinical outcomes, and they play a key role in immune suppression and tumor immune evasion [7,36].

Bone marrow Tregs exert both immunosuppressive and HSC-supportive functions, partly through the adenosine pathway and IL-10 production [37]. They maintain B-cell lymphopoiesis by controlling IL-7 production from stromal cells and play a crucial role in preventing autoimmunity, limiting chronic inflammation within the marrow and aiding engraftment during stem cell transplantation. Depletion of bone marrow Tregs increases HSC cycling, impairs stromal function, and reduces overall niche supportiveness [38,39].

MM cells, Tregs and their surrounding MSCs often upregulate ligands like PD-L1 or LAG-3 [40,41,42,43], which bind to the ‘off-switch’ receptors on T cells or dendritic cells (DCs) and induce a state of T-cell exhaustion, characterized by a loss of proliferative capacity and killing power [44]. TAMs contribute to the immunosuppressive microenvironment primarily through M2 polarization, a process driven by IL-10 and significantly enriched in myeloma bone marrow compared with healthy individuals [45]. M2-polarized TAMs activate STAT3 signaling in myeloma cells and suppress apoptosis by reducing caspase-3 cleavage.

### 2.3. Osteoclasts

Osteoblasts, derived from MSCs, and osteoclasts, originating from the hematopoietic lineage, are key components of the bone marrow microenvironment that coordinate bone remodeling through a tightly coupled process. These cells regulate each other’s activity via direct contact and signaling pathways while supporting HSC niches [46]. Expansion of osteoblasts in the endosteal niche is associated with increased hematopoietic populations [33], whereas osteoclasts contribute to homeostatic release and/or stress-induced mobilization of HSCs [47]. Osteoclasts also modulate the bone marrow microenvironment by regulating angiogenesis, influencing medullary niches by supporting osteoblast differentiation, maintaining functional niches and facilitating HSC homing. Importantly, distinct osteoclast subsets exert opposing effects on T-cell polarization: osteoclasts from normal bone marrow produce immunosuppressive cytokines such as IL-10 and TGF-β and promote Treg differentiation, whereas osteoclasts from an inflamed bone marrow drive TNF-α-producing CD4^+^ T cells [48].

Enhanced osteoclastogenesis and suppressed osteoblastic differentiation are key mechanisms in myeloma progression that create a permissive niche. Dysregulated cytokine production by myeloma cells and bone-resident cells creates a pathogenic feedback loop where bone destruction secretes cytokines to promote myeloma growth [49,50]. This loop is driven by an imbalance in the RANKL/OPG axis and cytokine signaling, in which myeloma progression favors increased RANKL expression and reduced osteoprotegerin levels [51]. Osteoclasts with activated RANKL remodel the endosteal niche to release dormant myeloma cells from the bone marrow [48]. Additionally, myeloma cells produce chemokines (MIP-1α, MIP-1β, SDF-1) that further enhance osteoclast activity while secreting osteoblast-inhibiting factors like Dickkopf-1 (DKK-1) [52]. They also participate in angiogenesis by producing VEGF and MMP9 along with osteopontin to increase blood vessels.

Osteoclasts suppress T-cell activation and proliferation through both antigen-dependent and antigen-independent mechanisms. They can function as antigen-presenting cells by expressing MHC class II and co-stimulatory molecules such as CD80 and CD86. Critically, osteoclasts in myeloma patients express elevated levels of multiple immune checkpoint molecules, including indoleamine 2,3-dioxygenase (IDO), PD-L1, HVEM, galectin-9, and CD200—often at higher levels than myeloma cells themselves—thereby contributing substantially to T-cell suppression and myeloma cell survival [48]. Activated T cells produce IFN-γ and CD40 ligand (CD40L), which induce IDO activity in osteoclasts. IDO depletes tryptophan from the tumor microenvironment, a metabolite essential for T-cell proliferation and survival, thereby suppressing T-cell responses to allogeneic, microbial, and T-cell receptor-mediated stimuli [53]. Osteoclasts can suppress T-cell activation induced by both non-specific stimuli (e.g., PHA) and physiologically relevant signals (CD3/CD28), indicating a broad and potent immunosuppressive capacity [54].

## 3. Modern Immunotherapeutic Approaches

The treatment paradigm in myeloma has undergone remarkable advances over the past decade, demonstrating prolonged survival and increasingly durable remissions. Novel therapies that harness and/or target the immune system have proven to improve patient outcomes. These next-generation cellular and immune-based therapies are also designed to address key mechanisms of disease progression and therapeutic resistance, including immune evasion driven by the bone marrow microenvironment, antigen loss or shedding, and immunoediting.

### 3.1. Cell Surface Proteins

**Figure 2:**
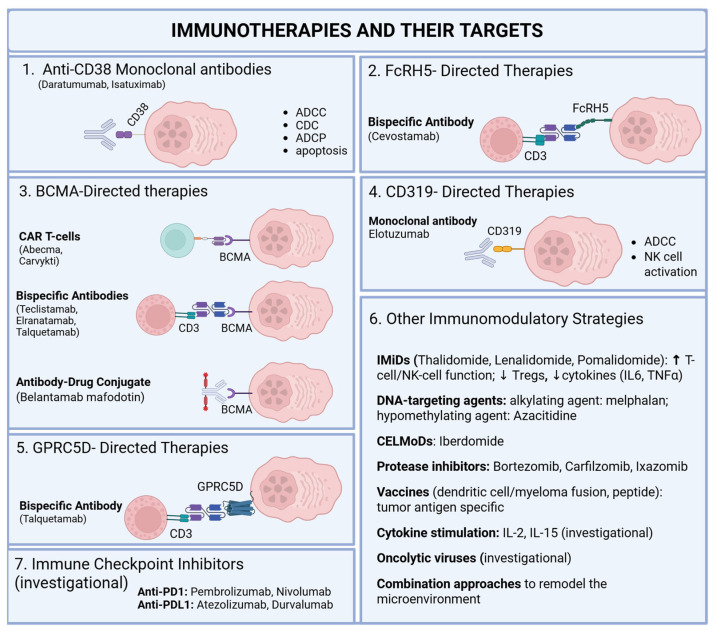
Immunotherapies and their respective targets in multiple myeloma. Cevostamab is in Phase II trial. Immune checkpoint inhibitors are FDA-approved and being investigated for treating MM. Other listed drugs are FDA-approved for treating MM. ↑ and ↓ indicate up- and down-regulation respectively.

The efficacy of targeted immune-related therapies hinges on the identification of surface antigens that are highly expressed on malignant plasma cells but minimally expressed on essential normal tissues for maximizing on-target and limiting off-target toxicity (Figure 2). BCMA, encoded by tumor necrosis factor receptor superfamily 17 (*TNFRSF17*), has emerged as the primary therapeutic target due to its critical role in promoting plasma cell survival and restricted expression on B-lineage cells [13,14]. Beyond BCMA, genomic and proteomic profiling has validated G protein-coupled receptor class C group 5 member D (GPRC5D) as a highly specific surface protein on plasma cells, leading to the first FDA-approved BiTE that targets it [15,16]. Additionally, Fc receptor-homolog 5 (FcRH5/*FCRL5*) and CD319/*SLAMF7* represent alternative lineage-specific targets that are currently being exploited for treating relapsed/refractory diseases (RRMM) [12,17,18,19]. Similarly, CD38 serves as a foundational target due to its uniform high expression as a multifunctional ectoenzyme on malignant plasma cells, which has enabled the widespread success of monoclonal antibody therapies despite its broader expression on other hematopoietic lineages [9,10].

### 3.2. Immune-Modulatory Drugs (IMiDs)

Immunomodulatory drugs (IMiDs) are thalidomide analogs (such as lenalidomide and pomalidomide) that exert anti-myeloma activity through multiple mechanisms, including inhibition of angiogenesis, direct tumor cytotoxicity, and enhancement of T-cell and NK-cell-mediated immune responses, making them one of the foundational agents in MM treatment [55]. Three generations of IMiDs are currently used in the treatment of MM: thalidomide (first generation), lenalidomide (second generation), and pomalidomide (third generation), all of which are FDA-approved [56]. Thalidomide received approval in 2006, marking the introduction of IMiDs into myeloma therapy. Lenalidomide is widely used across multiple disease settings, including newly diagnosed patients, transplant-ineligible individuals, RRMM patients, and as maintenance therapy following autologous stem cell transplantation. Pomalidomide, which is structurally related but more potent than lenalidomide, is primarily used in the relapsed/refractory setting, particularly in patients who have progressed on prior IMiD-based therapies [55]. In addition, emerging next-generation IMiDs like Mezigdomide and Iberdomide are demonstrating greater potency and efficiency than their predecessors and are rapidly moving through clinical trials [57,58].

Cereblon E3 ligase modulating drugs (CELMoDs) bind to cereblon (CRBN) with higher affinity than earlier IMiDs and induce more efficient degradation of *IKZF1* and *IKZF3*, even in settings of reduced CRBN expression, a known mechanism of IMiD resistance [59]. IMiDs bind to cereblon (CRBN), an E3 ubiquitin ligase component, which alters substrate specificity, recruits transcriptional factors *IKZF1* and *IKZF3* and triggers their ubiquitination and proteasomal degradation [8]. The downstream effects of cereblon modulation include reduced expression of *IRF4* and *MYC*, enhanced T- and NK-cell activation, and increased IL-2 production, ultimately leading to decreased myeloma cell survival [60]. Building on this mechanism, CELMoDs such as Iberdomide represent the next generation of IMiDs with encouraging clinical activity [58]. While both agents cause myelosuppression, CELMoDs have demonstrated improved tolerability with lower non-hematologic toxicity, along with enhanced T-cell activation (e.g., IFN-γ, IL-2) and partial rejuvenation of exhausted T cells [61]. CELMoDs have been observed to enhance antigen-specific CAR T-cell function and improve overall T-cell fitness [59]. Iberdomide is currently the most advanced CELMoD in Phase III clinical development and is being tested in combination regimens with other drugs like Elranatamab, as well as with daratumumab and dexamethasone for RRMM. Mezigdomide, another CELMoD in clinical trials, has demonstrated promising activity in heavily pretreated and IMiD-refractory patients [57,58,62,63].

### 3.3. Monoclonal Antibodies

Monoclonal antibodies (mAbs) are targeted immunotherapies that bind to specific antigens on myeloma cells and trigger an immune-mediated cytotoxic mechanism that can include antibody-dependent cellular toxicity, complement-dependent cytotoxicity and antibody-dependent cellular phagocytosis. These are highly effective and are often combined with steroids and other drugs like proteasome inhibitors and immunomodulatory agents. Currently, there are three FDA-approved mAbs for myeloma treatment—daratumumab [9,10], isatuximab [11] (anti-CD38), and elotuzumab [12] (anti-SLAMF7). These mAbs have significantly improved disease response rates and extended survival in MM patients.

CD38 is a transmembrane glycoprotein highly expressed on MM cells and on various immune cell subsets including T and B lymphocytes, natural killer (NK) cells, and monocytes. CD38 also functions as an ectoenzyme involved in NAD^+^ catabolism, contributing to immune regulation and local immunosuppression within the tumor microenvironment [64]. Daratumumab, the first fully human anti-CD38 IgG1 monoclonal antibody, was approved by the FDA in 2015 for the treatment of MM. Dara combined with lenalidomide and dexamethasone (DRd) or bortezomib and dexamethasone (DVd) is currently approved by the FDA and EMA for patients with at least one previous line of therapy. Its combination with bortezomib, melphalan, and prednisone (VMP) is also approved for newly diagnosed multiple myeloma (NDMM) patients who are ineligible for autologous stem cell transplantation (ASCT). However, despite its clinical efficacy, approximately 60% of the patients do progress, with some experiencing relapse^9^. A recent trial (MajesTEC-3) evaluating the combination of the bispecific antibody teclistamab with daratumumab in RRMM patients resulted in 83.4% PFS at 36 months, suggesting a promising future for applying anti-CD38-based combination therapies in treating relapsed patients [65].

### 3.4. Antibody–Drug Conjugates (ADCs)

Antibody–drug conjugates are biotherapeutics that consist of a tumor-targeting antibody linked to a potent cytotoxic payload via a chemical linker. The antibody directs the ADC to tumor-specific antigens, enabling targeted delivery of a highly potent cytotoxic compound, typically optimized for an appropriate drug-to-antibody ratio (DAR). Upon binding to the target antigen, the ADC is internalized through receptor-mediated endocytosis, after which the payload is released intracellularly and exerts its cytotoxic effect. Many ADCs have failed to advance past phase I clinical trials because balancing target specificity, payload potency, and tolerable toxicity has proven difficult [66,67].

Belantamab mafodotin, targeting BCMA, was the first ADC approved for RRMM as a monotherapy. It binds to BCMA on myeloma cells and delivers the cytotoxic payload monomethyl auristatin F (MMAF), a microtubule inhibitor, leading to cell cycle arrest and apoptosis after internalization [66]. However, its use as a single agent was limited by significant ocular toxicity, particularly keratopathy, and it faced challenges in Phase III trials. In late 2025, it was reapproved in combination with bortezomib and dexamethasone, where combination therapy improved efficacy while maintaining a manageable safety profile [68].

### 3.5. Chimeric Antigen Receptor (CAR) T Cells

Chimeric antigen receptor (CAR) T cells are autologous or allogeneic T cells genetically engineered to express synthetic receptors that recognize tumor-associated antigens independently of major histocompatibility complex (MHC)-mediated antigen presentation [69]. A typical CAR construct consists of an extracellular antigen-recognition domain, usually a single-chain variable fragment (scFv) that binds a tumor-specific antigen. This domain is linked via a transmembrane region—commonly derived from CD3, CD4, or CD8 molecules—to one or more intracellular co-stimulatory domains and then to the CD3ζ signaling domain, which mediates signal transduction and T-cell activation [70]. CAR-T cells mediate tumor killing through multiple pathways, including secretion of cytotoxic granules containing perforin and granzymes, production of pro-inflammatory cytokines like IFN-γ and TNF-α, and activation of the Fas/FasL pathway [71].

In myeloma, cell surface protein BCMA emerged as a primary target for CAR-Ts due to its ubiquitous expression on plasma cells [72]. Two FDA-approved BCMA-targeted CAR-T therapies include idecabtagene vicleucel (Abecma) (approved in 2021) and ciltacabtagene autoleucel (Carvykti) (approved in 2022), which have shown high response rates (70–90%+) in pretreated RRMM patients, with most CRS being grade1–2 [73]. CARTITUDE-4 is an ongoing randomized phase III trial evaluating Ciltacabtagene autoleucel versus standard of care in lenalidomide-exposed patients, demonstrating significant improvements in progression-free survival [73]. However, a significant proportion of the treated patients eventually relapse due to antigen escape or T-cell exhaustion and/or develop toxicities like CRS and neurotoxicity. Next-generation immunotherapies targeting GPRC5D, SLAMF7, and FcRH5 are being developed to overcome therapeutic resistance [72,74]. Armored CAR-T-cell therapy represents another emerging strategy designed specifically to overcome immune evasion by delivering additional genetic payloads that secrete cytokines (such as IL-12, IL-15) and/or block suppressive molecules (such as TGF-β) [75,76,77]. These modifications reduce pSmad2/3 signaling and T-cell exhaustion and, in turn, enhance CAR-T-cell persistence and function by altering its microenvironment. Novel molecules such as γ-secretase inhibitors can block BCMA cleavage and retain its expression on the MM cell surface to counter antigen escape for BCMA CAR-T [78]. Although these approaches are still in early-phase clinical trials, they hold considerable potential. In parallel, efforts are focused on dual-target CAR constructs, accelerated manufacturing platforms, and off-the-shelf CAR-NK cell therapies to improve accessibility, durability, and clinical scalability.

### 3.6. Bispecific T-Cell Engagers (BiTEs)

BiTEs are engineered antibodies designed to simultaneously bind CD3 on T cells and tumor-associated antigens, thereby redirecting cytotoxic T cells to eliminate cancer cells. They consist of two single-chain variable fragments (scFvs) connected by a flexible linker—one targeting CD3 and the other a tumor antigen—facilitating direct T-cell engagement, activation, and tumor cell killing [79]. These agents force an immunologic synapse between a T cell and a tumor cell, which pushes the inhibitory molecule CD45 out of the contact zone, allowing ZAP70 to move to the CD3 complex and activate T-cell signaling, ultimately leading to dose-dependent killing of the tumor cell. Current BiTE therapies in myeloma target three main antigens—BCMA, GPRC5D and FcRH5. Teclistamab, targeting BCMA/CD3, is the first FDA- and EMA-approved BiTE for RRMM and showed a 63% overall response rate at a maximum tolerated dose in phase I trials. Although CRS occurred in most patients, the majority of cases were low grade (Grade 1; ~50%) and typically resolved within 24 h [80], whereas Grade 3 or higher events were observed in only 0.6% of patients. Elranatamab is the second FDA-approved BCMA-directed CD3 bispecific antibody that demonstrated an overall response rate of approximately 58% in patients with RRMM, with 82% of responders maintaining their response at 9 months in clinical trials [81,82]. Other BCMA-targeted bispecific T-cell engagers include linvoseltamab and AMG 701, which are currently in various stages of clinical trials [82]. A GPRC5D-targeting BiTE, talquetamab, targets a transmembrane protein and showed 64–74% response rates in the MonumenTAL-1 trial, but it carries significant risks of CRS and neurological toxicity [83]. FcRH5-targeting BiTEs, such as cevostamab, target a B-cell-restricted protein retained in plasma cells. It has demonstrated manageable safety profiles in ongoing phase I studies, including those with prior BCMA therapy [84].

An emerging area of interest is the development of trispecific antibodies designed to address two major challenges—broader antigen coverage to reduce antigen escape and enhanced T-cell co-stimulation to limit T-cell exhaustion. These agents simultaneously target two tumor-associated antigens on myeloma cells, namely BCMA and GPRC5D, along with CD3 on T cells. Emerging trispecifics, such as ramantamig (JNJ-5322), target BCMA x GPRC5D x CD3 and are currently in Phase II clinical trials [85]. Preclinical studies have demonstrated potent cytotoxicity, robust T-cell activation and activity against both dual- and single-target-expressing cells. Other trispecific antibody in development includes ISB 2001, which targets BCMA, CD38, and CD3, and is currently in Phase I clinical trials. ISB 2001 has demonstrated early efficacy and durable responses in patients with BCMA-relapsed disease [86,87].

## 4. Resistance Mechanisms

The clinical success of immune-related therapies has revolutionized the treatment of hematological malignancies. However, despite high initial response rates, a significant proportion of patients experience treatment failure through various resistance mechanisms, which can be generally categorized into two categories: intrinsic factors that originate from the tumor cell itself; and extrinsic factors that originate from the surrounding environment or the immune system [64,88,89,90,91].

### 4.1. Intrinsic Resistance Mechanisms

#### 4.1.1. Innate Mechanisms

Intrinsic resistance involves the innate or acquired molecular qualities of the malignant cells that allow them to evade immune recognition or survive a cytotoxic attack. Innate antigen escape is observed in subgroups of MM cells that harbor genomic or transcriptomic abnormalities that enable immune evasion. For instance, aberrant splicing has been found in specific cytogenetic groups of MM, leading to the reduced expression of canonical *SLAMF7*/CD319 [92] as well as *FCRL5*/FCrH5 [93]. Another study indicates that 2.7% and 9% of NDMM patients have monoallelic inactivation of *TNFRSF17*/BCMA and GPRC5D, respectively [94]. As a result, T-cell activation is impaired due to the low expression level of antigens [95], contributing to immune escape.

#### 4.1.2. Acquired Mechanisms

Acquired antigen escape is more often found in patients who have received immune-related therapies. For instance, hypermethylation on the entire gene and promoter regions has been identified in patients who received anti-BCMA and anti-FCrH5 therapies [96]. Other genomic abnormalities such as structural variations or copy number loss are also reported to contribute to antigen escape [21,97,98,99,100]. Notably, with more extensive research, other intrinsic resistance mechanisms have been revealed. For instance, MM cells produce gamma-secretase that cleaves BCMA, making it soluble. The soluble BCMA further acts as a decoy and reduces the efficacy of anti-BCMA therapies [21,101,102]. Hypermethylation is also found at the promoter region of GPRC5D, thus acting as an epigenetic mechanism for silencing GPRC5D [20,103]. Combining gamma-secretase inhibitors with BCMA-targeted therapies in resistant patients has been seen to enhance therapeutic efficacy by increasing surface BCMA expression, improving response depth, and reprogramming the tumor microenvironment through effects on monocytes and macrophages [104,105]. Notably, MM cells can undergo sequential immunotherapies with different targets and develop resistance in penta-refractory MM patients (e.g., resistance to anti-BCMA, anti-GPRC5D and anti-CD38 therapies) [106].

MHC Class I and II molecules, as well as other molecules in the antigen-presenting machinery (APM), are critical in presenting antigens to T cells and triggering the immune response [107]. Malignant cells often downregulate these molecules or member genes of APM that are essential in the patient’s immune system to evade recognition by T cells [108]. Without a complete immune system, IMiDs targeting PD-1/PD-L1 become ineffective.

MM cells may overexpress survival proteins like BCL-2 or MCL-1 [109,110] for anti-apoptosis. Even if a T-cell successfully binds to the tumor and releases granzymes, the internal ‘self-destruct’ signal in the tumor cell is blocked by such overexpression, leading to a failure of cell death [111,112,113].

### 4.2. Extrinsic Resistance Mechanisms

As mentioned above, the MM bone marrow microenvironment comprises a dynamic and evolving ecosystem that provides a protective sanctuary for malignant plasma cells, enabling them to survive therapeutic pressure and evade immune detection [2,3]. This environment enables MM cells to generate a profoundly immunosuppressive milieu, characterized by expansion of MDSCs, Tregs, M2-polarized TAMs, as well as exhausted cytotoxic T and NK cells [114,115,116]. These immunosuppressive populations and the surrounding microenvironment not only fuel tumor proliferation and bone destruction but also establish a physical and biochemical barrier that attenuates the efficacy of current immunotherapies.

### 4.3. T-Cell/NK-Cell Exhaustion

The persistent immunosuppressive myeloma microenvironment ultimately drives immune exhaustion, characterized by chronic antigen stimulation, immune dysfunction, and sustained engagement of inhibitory receptors such as PD-1, TIGIT, TIM-3, LAG-3 and granzyme B (GZMB). Co-expression of these multiple inhibitory molecules has been associated with increased T-cell exhaustion and poor tumor control in both animal models and human studies [22,34,117,118,119]. These molecules are commonly referred to as exhaustion markers, as they are predominantly induced following T-cell receptor (TCR) stimulation and are associated with activated T-cell states. Chronic T-cell receptor (TCR) signaling induces the transcription factor TOX (thymocyte selection-associated HMG box protein), which regulates inhibitory receptor expression, chromatin remodeling, and maintenance of exhausted T-cell states [120]. As precursor exhausted T cells progress toward terminal exhaustion, they lose expression of TCF7, a transcription factor essential for T-cell maintenance and maturation; accordingly, TCF7-deficient CD8^+^ T cells fail to sustain long-term function under chronic antigen stimulation [120,121]. Therapies such as CAR-T cells and T-cell engagers rely on robust T-cell function; therefore, an exhausted immune state can limit their efficacy and contribute to therapeutic resistance. In the exhaustive state, T-cell distribution and function are impaired, including reduced frequencies of CD4^+^ and CD8^+^ T cells and imbalanced Th1/Th2 ratios, leading to compromised T-cell responses [122]. Multiple studies have also confirmed that T-cell exhaustion is associated with early myeloma relapse in ASCT patients through exhaustion signatures like upregulation of PD1/TIGIT and downregulation of CD28 in T cells [23,98,123,124,125]. Some studies also describe a state of T-cell immunosenescence driven by both aging and the accumulation of senescent T cells. Senescent T cells are characterized by reduced apoptotic capacity, downregulation of CD28, and upregulation of CD57 and PD-1, indicating these T cells are not exhausted yet [43,126]. T-cell senescence is primarily associated with aging, cellular stress, DNA damage, and loss of proliferative capacity, whereas T-cell exhaustion is driven by persistent antigen and T-cell receptor (TCR) stimulation and may remain partially reversible, particularly in progenitor-like exhausted states. In cancer, these states frequently coexist and overlap, resulting in intersecting dysfunctional immune programs [127,128].

### 4.4. Immunosuppressive Cells

The tumor microenvironment (TME) in MM is characterized by an expansion of immunosuppressive cell populations that produce inhibitory factors, limiting immune infiltration and activation of malignant plasma cells [114,115,116]. As the disease progresses from precursor stages to active MM, there is a significant increase in MDSCs and Tregs, which secrete inhibitory cytokines like IL-6, IL-10 and TGF-β, which further downregulate the activities of Th1, Th17, macrophages and dendritic cells (DC) [7,129]. Moreover, Tregs can inhibit these cells via direct interaction. All these factors can undermine the efficacy of therapies such as CAR-T or BiTEs. Tregs and Bregs further contribute by producing immunosuppressive factors that inhibit effective immune surveillance. These cells increase the expression of CD38 and are targeted by CD38-directed antibodies like daratumumab and isatuximab. These treatments can restore the immune microenvironment by reducing CD38+ Tregs, MDSCs, and Bregs and promoting the expansion of CD4+ and CD8+ T cells, which likely contributes to the clinical success of combinations such as daratumumab plus teclistamab in myeloma patients. Activated Tregs accumulate near the malignant MM cells and form a protective niche, facilitating the initial blood dissemination of MM cells in the bone marrow [130]. Endogenous NK and CD8+ T cells are affected/blocked by these cells, contributing to immune evasion. Treg proliferation is stimulated by mechanisms like APRIL signaling via the TACI receptor along with the production of immune-suppressive markers like Foxp3, IL-10, and PD-L1 [131]. MSCs and cancer-associated fibroblasts (CAFs) can interact with the extracellular matrix and create a physical barrier that prevents immune cells from physically reaching the tumor nests [132,133]. CD123, the α-chain of the IL-3 receptor, is abundantly expressed on MDSCs but is unlikely to be effective as a monotherapy target due to its expression on other immune cell populations and the presence of compensatory pathways within the microenvironment. However, bispecific antibodies targeting both myeloma cells and MDSCs may redirect T-cell cytotoxicity in an MHC-independent manner [134]. CD123 × CD3 bispecific antibodies such as Vibecotamab, MGD024, and APVO436 are actively being tested in early clinical trials (especially in AML/MDS/CMML) in combination with azacitidine [132,135,136].

TAM-mediated STAT3 activation also promotes resistance to therapy, including partial resistance to bortezomib through JAK2/STAT3 signaling [137]. Targeting this pathway—via inhibition of IL-10/IL-10R signaling or treatment with JAK2 inhibitors such as ruxolitinib—has been shown to re-sensitize myeloma cells to therapy in vitro and in vivo [138].

PD-L1, which is overexpressed on osteoclasts during osteoclastogenesis, engages with PD1 on T cells to inhibit their proliferation and cytotoxic functions. Osteoclasts indirectly induce PD-L1 upregulation on myeloma cells by enhancing IFN-γ secretion from T cells, leading to downstream immune suppression [139]. Co-culture of osteoclasts with CD4^+^ and CD8^+^ T cells increases PD-1 expression on T cells and suppresses their anti-myeloma cytotoxic activity [140]. Treatment with anti-PD-L1 monoclonal antibodies and IDO inhibitors has been shown to partially restore T-cell responses suppressed by osteoclasts in myeloma [139]. These findings have also been validated in co-culture studies using patient-derived autologous cells, including plasmacytoid dendritic cells, NK cells, T cells, and myeloma cells. In this setting, inhibition of the kynurenine pathway enzyme, kynurenine-3-monooxygenase (KMO), which is upregulated in dendritic cells, reactivates dendritic cell function and enhances cytolytic activity of both cytotoxic T lymphocytes (CTLs) and NK cells against myeloma cells [141].

### 4.5. Metabolic Competition

The success of CAR-T and BiTE therapies is not solely determined by the genetic ‘rewiring’ of the T-cell, but also by its ability to maintain metabolic fitness and effector function within a nutrient-depleted tumor microenvironment, which occurs because both malignant cells and immune cells rely on the same restricted pool of nutrients to fuel their rapid proliferation. For instance, glycolysis can be induced by both toll-like receptor (TLR)-induced signaling to boost mammalian target of rapamycin (mTOR) [142] and by activated NK cells [143,144]. Since increased glycolysis (the Warburg effect) is also a hallmark of MM cells [145], the malignant cells exhibit high rates of glucose consumption, leading to nutrient depletion that limits immune cell metabolism. Furthermore, the tumor’s metabolic byproduct, lactic acid, creates a severely acidic environment that blunts T-cell signaling and promotes a state of anergy. Beyond glucose, the depletion of critical amino acids—such as glutamine and tryptophan—by tumor-expressed enzymes like IDO1 further handicaps the immune response while simultaneously recruiting Tregs. This metabolic deprivation effectively forces the therapeutic T cells into a state of functional paralysis, resulting in impaired cytotoxic activity despite adequate antigen recognition.

Additionally, adhesion of myeloma cells to MSCs, together with niche-derived signaling, promotes cell adhesion-mediated drug resistance (CAM-DR) through upregulation of adhesion molecules such as VCAM-1, ICAM-1, and CD40 [146,147]. This interaction plays a significant role in drug resistance as it helps the myeloma cells to maintain normal or even increased OXPHOS and mitochondrial metabolism compared to normal cells. MSCs protect myeloma cells by donating functional mitochondria via tunneling nanotubes (TNTs), restoring oxidative phosphorylation (OXPHOS), ATP production, and redox balance [148]. This process increases MM cell survival and ATP levels, particularly under chemotherapeutic stress [149,150]. This mechanism is CD38-dependent and supported by the CXCL12/CXCR4 axis, resulting in an approximately 1.5-fold increase in mitochondrial ATP production [151,152]. Critically, this mitochondrial transfer is bidirectional and proportional to drug concentration, providing MM cells with metabolic flexibility and chemoresistance, and plays a crucial role in the development of drug resistance [153]. The transfer can be observed both in vitro and in vivo [149,151,154,155], suggesting it is a robust protective mechanism in the MM microenvironment. This is particularly important in extramedullary myeloma, in which myeloma cells lose their bone marrow dependency and migrate outside the bone marrow niche to soft tissues and organs. It is associated with worse prognosis and drug resistance. These complex interactions with stromal cells, immune cells and endothelial cells help downregulate adhesion markers like CD56 and VLA-4 and activate EMT-like pathways [156].

### 4.6. T-Cell/NK-Cell Fitness

The fitness level of T cells is critical for maintaining efficacy in most immune-related therapies. An exhausted immune system will not carry out adequate immunity against MM cells [44]. Molecules such as alkylating agents that are standard therapeutic options in MM often generate high levels of reactive oxygen species (ROS) that induce a state called ‘T-cell senescence’ [157], which is characterized by shortened telomeres and a permanent arrest in the cell cycle [158,159], leading to a lack of proliferative capacity required for a robust anti-tumor response. The transcription factor TOX, co-expressed with PD-1 and TIGIT, is overexpressed in CD8^+^ T cells from the bone marrow of myeloma patients compared with peripheral blood [160,161]. These dysfunctional phenotypes are associated with reduced T-cell proliferation and impaired cytokine production. Most PD1+ CD8+ T cells show other exhaustion markers, like TIM3 and/or LAG3. TIGIT binds to its ligand CD155 and CD112 on myeloma and bone marrow mesenchymal cells, in turn activating the CD155/TIGIT signaling pathway, resulting in reduced TNF-α and IFN-γ production, decreased CD107a expression indicative of impaired cytotoxicity, and diminished T-cell proliferative capacity [22,118]. Other factors such as aging and chronic infections can also drive the accumulation of senescent T cells [162]. The memory phenotype of T cells is perhaps the single most critical biological factor determining whether immune-related therapies like CAR-T or BiTEs result in a temporary response or a permanent cure [163], which determines when the disease will relapse. Consequently, applying these therapies in earlier treatments has been increasingly considered to harvest ‘fit’ T cells before they irreversibly become senescent or exhausted [164,165,166].

Natural killer (NK) cells are additional cytotoxic effector cells affected by myeloma, with higher NK-cell activity associated with improved survival and prognosis, and reduced activity linked to advanced disease stage [119,167,168]. Unlike T cells, NK cells recognize and eliminate abnormal cells without prior sensitization and in an MHC-independent manner. An activating receptor named killer cell lectin-like receptor subfamily K member 1 (KLRK1), also known as NKG2D, mediates anti-tumor response through a process called ‘induced-self recognition’. Its expression can be induced by cellular stress, infection and/or malignant transformation. NK-cell fratricide is typically triggered following NKG2D-mediated activation as a regulatory mechanism, where activated NK cells kill not only target cells but also other NK cells through the NKG2D-induced perforin pathway in vitro and in vivo [169]. Hypoxia, lactate, and PGE2 reduce NK cell cytotoxicity in MM; this impairment can be partially reversed by daratumumab, which enhances NK-cell function by targeting CD38^+^ myeloma cells and increasing susceptibility to CD16-mediated antibody-dependent cellular cytotoxicity (ADCC), highlighting a potential therapeutic strategy [170,171].

There are two distinct fratricide pathways—(1) hypoxia-induced and (2) ligand-mediated. Hypoxia downregulates NK cell activity by downregulating NKG2D, natural cytotoxicity receptors (NKp46, NKp30, and NKp44) and other cytotoxic effectors such as intracellular perforin and granzyme B levels, thereby reducing myeloma cell killing in an oxygen-dependent manner [172,173]. This impaired cytotoxicity can be rescued by IL-2-mediated activation [173]. NK cells can also rapidly acquire NKG2D receptors by a process called trogocytosis within 10 mins of cell–cell contact [169]. This process requires direct contact and depends on NKG2D signaling via the DAP10/DAP12 adaptor proteins, clathrin-dependent endocytosis, and activation of PI3K and Syk signaling pathways [174,175]. Once acquired, NK cells display NKG2D ligands on their surface, leading to their recognition and elimination by fellow NK cells. These two pathways end up suppressing NK function and adding immune exhaustion and leeway for MM cells.

## 5. Toxicities

**Figure 3:**
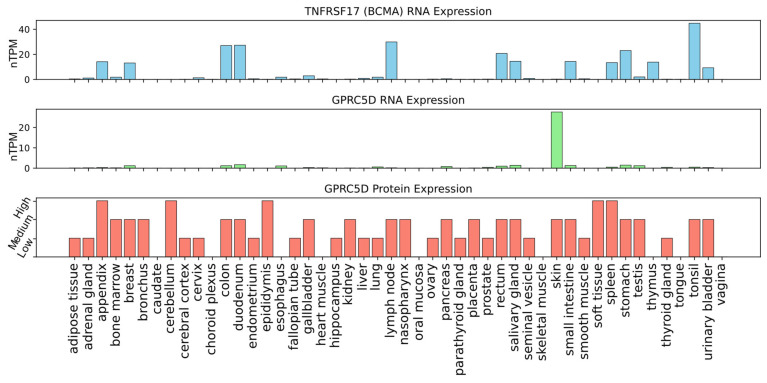
RNA and protein expression levels of immunotherapy targets in healthy organs. Protein expression of BCMA is absent in the Human Protein Atlas.

Although the emergence of novel immunotherapies has significantly improved the survival of patients with multiple myeloma (MM), these agents are associated with unique and sometimes severe toxicities, presenting clinical challenges that require careful management to maintain therapeutic efficacy. In hematological malignancies, the most common acute toxicities associated with T-cell-redirecting therapies are CRS (cytokine release syndrome) and ICANS (immune effector cell-associated neurotoxicity syndrome) [176,177]. CRS is a systemic inflammatory response triggered by a rapid and abrupt increase in cytokines (primarily IL-6) in the bloodstream upon T-cell activation in T-cell-redirecting therapies [178]. Its symptoms can scale from mild (e.g., fever, fatigue, headache and pain) up to fatal (e.g., multi-organ failure) [178,179]. Tocilizumab (anti-IL-6) and corticosteroids are considered options for managing CRS [180] and have been shown to reduce the toxicity when given prophylactically [181]. ICANS is a potential neurological side effect resulting from high levels of immune activity, causing inflammation in the brain. Both BiTEs and CAR-T therapy trigger ICANS through a massive release of cytokines that lead to an immune response in the brain [182]. The prophylactic administration of the IL-1 receptor antagonist anakinra or low-dose dexamethasone has demonstrated efficacy in stabilizing the blood–brain barrier and reducing the incidence of high-grade ICANS without compromising the long-term anti-tumor activity of the CAR-T cells [182,183]. Other toxicities, such as ocular adverse events, are associated with the payload from antibody–drug conjugates (ADCs) such as BLENREP [184]. The expression of antigens on healthy organs is also associated with specific adverse events, such as nausea (24%~28%), diarrhea (12%~30%), constipation (13%~22%) and vomiting (5%) in BCMA therapies [25,101,185], for which expression can be found in the colon and intestine (Figure 3), as well as skin-related (67%), nail-related (57%~65%), rash-related (18%~47%), dysgeusia (53%~67%) and dry mouth (6%~30%) events in GPRC5D therapies [15,186], for which expression can be found in skin (Figure 3). Additionally, infection remains a leading cause of non-relapsed mortality in patients who received immune-related therapies. Patients are particularly susceptible to pathogens such as bacterial, fungal and viral infections [187,188].

## 6. Conclusions

Targeted immunotherapies, including CAR-T cells, BiTEs, and ADCs, have significantly improved clinical outcomes for patients with multiple myeloma. By targeting plasma cell-specific antigens such as BCMA, GPRC5D, and CD38, these modalities achieve robust initial response rates even in heavily pretreated populations. However, resistance cases are still frequently reported, as the durability of these therapies is frequently limited by both intrinsic tumor evolution and extrinsic microenvironmental pressures. Proactively addressing mechanisms such as antigen escape, metabolic competition, and T-cell exhaustion is essential for designing next-generation constructs and rational combination strategies and for improving long-term patient outcomes. Fortunately, novel approaches such as trispecific therapies emerge as promising ways to reduce antigen escape due to the simultaneous engagement of two distinct tumor antigens. In parallel, the continuous identification of novel targets [93] (e.g., subtype-specific antigens) and the subsequent development of therapeutic options further enrich the arsenal of immunotherapies. Other novel approaches such as ‘armored CAR-T’ cells or combinational therapies are designed to alter the ‘hostile’ tumor microenvironment to reduce extrinsic resistance. These next-generation approaches hold immense promise for increasing patients’ response rate and prolonging their survival. Nonetheless, MM is notoriously heterogeneous. Unique combinations of primary and secondary genomic events drive the expression of novel, subtype-specific antigens. Characterizing these distinct antigenic profiles will be a critical task in the future. Concurrently, overcoming extrinsic resistance mechanisms by the tumor microenvironment is equally important and urgent. Developing metabolically robust ‘armored CAR-T’ cells capable of surviving and functioning in the nutrient-depleted and acidic local environment will be essential for enhancing T-cell persistence. Crucially, because two resistance mechanisms could dynamically interplay in patients, a systematic evaluation of patients’ immune-response landscapes is a prerequisite when designing treatment regimens.

A central component of the immune landscape is immune effector cells, which can be both quantitatively and qualitatively impaired by prior therapies; the strategic sequencing of immunotherapies is critical for maximizing patient outcomes. For instance, continuous activation of T cells by bispecific T-cell engagers (TCEs) increases the expression of inhibitory receptors, which can compromise the durability of responses to subsequent cellular therapies [189]. Consequently, evaluating the dynamic immune landscape and incorporating sufficient treatment-free intervals to allow for T-cell recovery are essential steps when designing sequential immunotherapeutic strategies. Moreover, the impact of effector cell fitness on therapeutic efficacy prompts a critical re-evaluation of the optimal timing for introducing immune therapies within the treatment paradigm. These cellular and T-cell-redirecting therapies rely on a host immune system that may have already been rendered dysfunctional by chronic disease burden and cumulative exposure to DNA-damaging agents, such as alkylating drugs. Because the memory phenotype and overall fitness of T cells largely determine the potential for a durable response, shifting these modalities to earlier lines of therapy is increasingly being considered due to a healthier, less-exhausted immune system in patients treated with few lines of therapy versus heavily pretreated patients.

Ultimately, the interplay between tumor biology and immune fitness makes patients’ response prediction far more complex than expected. Following relapses on an initial targeted therapy, malignant plasma cells frequently exhibit acquired resistance mechanisms, such as genomic structural variations, copy number loss, or promoter hypermethylation, leading to diminished or absent antigen expression. It is reasonable to believe that re-challenging a patient with another immune-directed therapy against the same targets—such as sequential BCMA-targeted modalities—may yield diminishing returns due to impaired target recognition. However, emerging clinical data indicates that patient responses are far more complex and unpredictable than we anticipated. For instance, retreating patients with a second therapy directed at the same target, such as sequential BCMA-directed modalities, can still be highly effective despite the potential presence of resistant subclones [190]. These findings suggest that while acquired immune evasion is a critical factor, the specific mechanisms of resistance, the timing of relapse, and the duration of the initial response deeply influence subsequent efficacy. This unpredictability underscores the necessity of comprehensive molecular profiling at relapse while also highlighting that previously targeted antigens may still present viable therapeutic vulnerabilities.

## Data Availability

No new data was generated in this study. All data used in this study is cited in the article.
